# Role of the Vasohibin Family in the Regulation of Fetoplacental Vascularization and Syncytiotrophoblast Formation

**DOI:** 10.1371/journal.pone.0104728

**Published:** 2014-09-03

**Authors:** Kaori Suenaga, Shuji Kitahara, Yasuhiro Suzuki, Miho Kobayashi, Sachiko Horie, Junichi Sugawara, Nobuo Yaegashi, Yasufumi Sato

**Affiliations:** 1 Department of Vascular Biology, Institute of Development, Aging, and Cancer, Tohoku University, Aoba-ku, Sendai, Miyagi, Japan; 2 Department of Obstetrics & Gynecology, Tohoku University School of Medicine, Aoba-ku, Sendai, Miyagi, Japan; 3 Department of Anatomy and Developmental Biology, Tokyo Women's Medical University, Shinjuku-ku, Tokyo, Japan; 4 Tohoku Medical Megabank Organization, Tohoku University, Aobaku, Sendai, Miyagi, Japan; Feinberg Cardiovascular Research Institute, Northwestern University, United States of America

## Abstract

Vasohibin-1 (VASH1) and vasohibin-2 (VASH2), the 2 members of the vasohibin family, have been identified as novel regulators of angiogenesis. VASH1 ceases angiogenesis, whereas VASH2 stimulates sprouting. Here we characterized their functional role in the placenta. Immunohistochemical analysis of human placental tissue clarified their distinctive localization; VASH1 in endothelial cells and VASH2 in trophoblasts. We then used a mouse model to explore their function. Wild-type, *Vash1^(−/−)^*, and *Vash2^(−/−)^* mice on a C57BL6 background were used in their first pregnancy. As expected, the fetal vascular area was increased in the *Vash1^(−/−)^* mice, whereas it was decreased in the *Vash2^(−/−)^* mice relative to wild-type. In addition, we noticed that the *Vash2^(−/−)^* mice at 18.5dpc displayed thinner villi of the labyrinth and larger maternal lacunae. Careful observation by an electron microscopy revealed that the syncytiotrophoblast formation was defective in the *Vash2^(−/−)^* mice. To test the possible involvement of VASH2 in the syncytiotrophoblast formation, we examined the fusion of BeWo cells, a human trophoblastoid choriocarcinoma cell line. The forskolin treatment induced the fusion of BeWo cells, and the knockdown of VASH2 expression significantly inhibited this cell fusion. Conversely, the overexpression of VASH2 by the infection with adenovirus vector encoding *human VASH2* gene significantly increased the fusion of BeWo cells. Glial cell missing-1 and endogenous retrovirus envelope glycoprotein Syncytin 1 and Syncytin 2 are known to be involved in the fusion of trophoblasts. However, VASH2 did not alter their expression in BeWo cells. These results indicate that VASH1 and VASH2 showed distinctive localization and opposing function on the fetoplacental vascularization. Moreover, our study shows for the first time that VASH2 expressed in trophoblasts is involved in the regulation of cell fusion for syncytiotrophoblast formation.

## Introduction

The placenta is an organ that connects the fetus to the maternal uterine wall, and it starts to develop upon implantation of the blastocyst into the maternal endometrium. The outer layer of the blastocyst becomes the trophoblast, which forms the outer layer of the placenta. Subsequently, this layer of trophoblast cells is subdivided into the cytotrophoblast and syncytiotrophoblast layers. Cytotrophoblasts proliferate and invade the endometrial tissue to form placental villi. The multinucleate cell layer of syncytiotrophoblasts is formed by the cell fusion of cytotrophoblasts and covers the entire surface area of the placenta [1].

The placenta is a highly vascularized organ that allows for nutrient uptake, waste elimination, and gas exchange for the developing fetus. The placental circulation brings the fetal and maternal vascular systems into close relationship, and multiple steps of vascular development and/or remodeling on both fetal and maternal sides are required for acquisition of this relationship. These steps include (i) invasion by trophoblast cells, (ii) vascularization within the trophoblast layer to establish and maintain the fetoplacental vasculature, and (iii) subsequent maternal vascular remodeling to gain the uteroplacental circulation [2], [3].

The fetoplacental vasculature is formed by vasculogenesis and angiogenesis, and multiple regulatory systems are reported to regulate these processes. They include the vascular endothelial growth factor (VEGF)/VEGF receptor (VEGFR) system, angiopoietin/TIE receptor system, platelet-derived growth factor (PDGF)/PDGF receptor system, and transforming growth factor ß (TGF- ß)/TGF- ß receptor system [4]. Among them, VEGF-A, a prototype of the VEGF family, is considered to be the most important factor that promotes vasculogenesis and angiogenesis in the entire body including the placenta. VEGF-A is intensively expressed in cytotrophoblasts, particularly in the early developmental stage of the placenta. VEGFR2 is the major mediator of VEGF-A-driven responses in vascular ECs. VEGFR1, on the other hand, has higher affinity for VEGF-A but weaker tyrosine kinase activity. Soluble VEGFR1 (sVEGFR1), a splicing variant of VEGFR1, is highly expressed in trophoblasts and traps VEGF-A by acting as a decoy receptor. Placenta growth factor (PlGF), another member of the VEGF family, is also highly expressed in trophoblasts, but the function of PlGF in the development of placenta has not yet been well characterized [5], [6].

Vasohibin-1 (VASH1) was isolated as a negative-feedback regulator of angiogenesis induced in ECs by angiogenesis stimulators such as VEGF and FGF-2 [7]. Subsequently, a gene homologous to VASH1 was identified and named vasohibin-2 (VASH2) [8]. The amino acid sequence of the human VASH2 protein is 52.5% homologous to that of human VASH1, and both VASH1 and VASH2 are highly conserved among species [9]. Although vasohibins lack classical signal sequence for their secretion, they bind to small vasohibin binding protein (SVBP) within a cell and that facilitates the secretion of vasohibins [10].

Expression and function of VASH1 and VASH2 have been examined by the use of hypoxia-induced subcutaneous angiogenesis in mice, and the results revealed that VASH1 is mainly expressed in ECs in the termination zone to halt angiogenesis, whereas VASH2 is mainly expressed in mononuclear cells mobilized from the bone marrow in the sprouting front to stimulate angiogenesis [11]. Thus, these 2 vasohibin family members regulate angiogenesis in a contradictory manner.

As mentioned above, angiogenesis regulators are involved in the regulation of placental morphogenesis, but little is known about the function of vasohibin family in this regulation. Hence, in this present study we characterized the localization of these vasohibins in the human placenta and their expression and function in the murine placenta.

## Materials and Methods

### Immunohistochemistry for human placenta

The Ethics Committee at Tohoku University approved this study. Human placenta was obtained from normal pregnant women (38∼40 weeks of gestation) who provided their written consent to participate in this study at Tohoku University Hospital. The Ethics Committee at Tohoku University approved this consent procedure. Samples were dissected into 2×2×2 cm cubes, fixed at 4°C in 4% paraformaldehyde (Wako, Osaka, Japan) for 2 days, and then embedded in paraffin. Sections (5 µm) were prepared and then deparaffinized, after which endogenous peroxidase activity was blocked by immersion in 3% H_2_O_2_ (Santoku, Tokyo, Japan)/methanol for 10 min. The sections were then autoclaved for 5 min at 121°C in Target Retrieval Solution, pH 6 (Dako, CA) and then blocked for 30 min with 1% bovine serum albumin (BSA, Sigma-Aldrich, MO) diluted in phosphate-buffered saline (PBS) containing 0.1% Tween20 (Sigma-Aldrich). Next, the sections were incubated overnight at 4°C with 1st antibodies, anti-human VASH1 mAb (4E12) [7] and anti-human VASH2 mAb (5E3) [8]. On the next day, they were washed with PBS and then incubated with N-histofine simple stain Mouse Max PO (Nichirei, Tokyo, Japan) for 30 min. After having been washed with PBS, the sites of immunoreactivity in the sections were visualized with diaminobenzidine (DAB tablet, Wako).

### Animal model of placentation

All of the animal studies were approved by the Center for Laboratory Animal Research of Tohoku University. Nine- to twelve-week-old wild-type (WT), *Vash1^(−/−)^* or *Vash2^(−/−)^* mice on a C57BL/6 background [9] were mated and used in their first pregnancy for the present study. The day when the vaginal plug was observed was defined as day 0.5 of gestation. Mice were sacrificed on 12, 16, and 18 day post-coitum (dpc). Both fetal and maternal body and organ weights were recorded. Some of the mice were snap frozen in liquid nitrogen for subsequent analysis.

### Immunohistochemistry of mouse placenta

Intravascular perfusion with fluorescein lycopersicon esculentum (TOMATO) lectin (Vector Laboratories, CA) was performed to label vessels for maternal blood circulation as described previously [12]. Briefly, mice were injected intravenously with 100 µl of TOMATO lectin; and 10 min later their thorax was opened and the aorta perfused with 4% PFA (Wako) in 0.1 M PBS at a pressure of 100–120 mm Hg for 5 min, followed by perfusion with PBS for 5 min via the left ventricle. After perfusion, the tissues were processed for subsequent analyses. These tissues were kept overnight at 4°C in 30% sucrose in PBS, and the next day they were embedded in O.C.T compound (Sakura Finetek, CA). Subsequently, they were frozen and stored at −80°C in a deep freezer until use. Cryosections (20 µm) were prepared and washed in PBS, after which the endogenous peroxidase activity was quenched for 10 min by immersion in 3% H_2_O_2_/methanol. The blocking of non-specific binding sites was performed for 30 min by incubation in PBS containing 1% BSA and 0.1% Tween20. First antibodies, which were purified rat anti-mouse CD31 (BD Biosciences, CA), biotinylatead anti-mouse HAI-1 antibody (R&D systems, MN) and anti-type IV collagen (ab6586, Abcam, MA) were diluted 1∶200 in PBS containing 1% BSA and 0.1% Tween20. After removal of the blocking buffer, the sections were reacted with the 1st antibodies at 4°C overnight. On the next day, after a wash with PBS the sections were incubated for 30 min at RT with fluorescent secondary antibodies, i.e., Alexa Fluor 633-conjugated goat anti-rat IgG (Molecular Probes, Eugene, OR) and Alexa Fluor 555-conjugated donkey anti-rabbit IgG (Molecular Probes), which had been diluted 1∶200 in PBS. Finally, the specimens were mounted with fluorescent mounting medium (Dako, CA). Ten fields per section were randomly selected and observed under a fluorescence microscope, and the fetal vascular area was calculated by using software (BZ-9000, Keyence, Osaka, Japan), and the maternal vascular area was calculated by using imageJ 1.48v, an open source Java image processing program.

### Enzyme linked immunosorbent assay (ELISA)

Serum samples were collected for the determination of the levels of murine VEGF, soluble VEGFR1, and PlGF. ELISA kits for murine VEGF, solubleVEGFR1, and PlGF were purchased from R&D Systems. ELISA was performed according to the manufacturer's instructions.

### Transmission electron microscopy

After perfusion with 4% PFA, some samples of the mouse placenta with uterus were cut into small blocks and incubated in 2% glutaraldehyde in 0.1 M PB for 2 hours. The samples were subsequently incubated with a 1% solution of OsO_4_ for 1 hour at 4°C, dehydrated by passage through a graded series of ethanol followed by propylene oxide, and embedded in epoxy resin. Ultrathin sections (70 nm) were stained with lead citrate and examined with an H-7000 electron microscope (Hitachi, Tokyo, Japan).

### Reverse transcriptase-polymerase chain reaction (RT-PCR)

Total RNA was prepared from the placenta of 12.5-, 16.5-, and 18.5-dpc mice by using ISOGEN (Nippon Gene, Toyama, Japan) according to the manufacturer's instructions. Single-stranded cDNA was synthesized by using ReverTra Ace (TOYOBO, Osaka, Japan). RT-PCR was performed with a thermal cycler system (CFX-96 Real-Time system, C1000 Thermal Cycler, Bio-Rad, Tokyo, Japan) and SYBR Premix Ex Taq (TaKaRa). The primer pairs used were as follow: mouse GAPDH, 5′-TGAACGGGAAGCTCACTGG-3′ (forward) and 5′-TCCACCACCCTGTTGCTGTA-3′ (reverse); mouse Gcm-1, 5′-TCCAACTCCTTACGGATGAA-3′ (forward) and 5′- GGGCGTTAGCTATTAAAGGTG-3′ (reverse); Syncytin-B, 5′-TCTCACTGGCACTTCATTCC-3′ (forward) and 5′- TCAGGTTATGAGGTGAGAGG-3′ (reverse); Syncytin-A, 5′- TTGGTTGACTTCCCTCATGG-3′ (forward) and 5′-AGCAGAAGGATCTTGTCCAC -3′ (reverse).

### Cell-cell fusion analysis in vitro

Cells of the human choriocarcinoma cell line BeWo, obtained from RIKEN BioResource Center (Ibaraki, Japan), were cultured in Ham's F10 (Sigma-Aldrich) supplemented with 10% fetal bovine serum(FBS; BioWest S.A.S, Nuaillé, France)and treated with 20 µM forskolin (FK, Sigma-Aldrich) or vehicle (dimethyl sulfoxide; DMSO, Sigma-Aldrich) for 48 hours. Thereafter, immunohistochemistry was performed to detect cell-cell fusion. The cells were first fixed with 3% formaldehyde (Wako) for 10 min at RT. They were then incubated for 10 min with 0.3% Triton-X in TBS and subsequently washed with Tris- buffered saline (TBS, TaKaRa). After blockage of non-specific binding sites for 30 min with 5% BSA in TBS anti-E cadherin at 10 µg/ml (M108, TaKaRa), as 1st antibody, was applied overnight at 4°C. On the next day, the 2nd antibody reaction was performed for 45 min at RT with Alexa 488-conjugated rat IgG (Molecular Probes) at a 1∶200 dilution and DAPI at a 1∶10,000 dilutions (Invitrogen Life Technologies, Carlsbad, CA). The cells in 10 randomly selected fields per culture dish were observed with a fluorescence microscope (BZ-9000, Keyence) at 400-power magnification. Cell fusion was detected by the loss of E-cadherin between cells, and the number of fused cells (syncytia) was counted. Adobe Photoshop CS6 was used to calculate the fusion index: [(N-S)/T]×100%.(N; the number of nuclei in the syncytia, S; the number of syncytia, T; the total number of nuclei counted) [13], [14].

For the knockdown of VASH2, BeWo cells were transfected with a non-targeting control small interfering RNA (siRNA) or human VASH2 siRNA by using Lipofectamine RNAiMax (Invitrogen) with Opti-MEM at a final concentration of 25 nmol/L according to the manufacturer's instructions. The siRNA used for human VASH2, were designed and purchased from Invitrogen Life Technologies, and their sequences was 5′-CACUCUGAAUGAAGUGGGCUAUCAA -3′ (sense) and 5′-UUGAUAGCCCACUUCAUUCAGAGUG -3′ (antisense). Non-specific StealthRNAi Negative Control Medium GC Duplex #2 was used as control, was also purchased from Invitrogen. After a 24-h incubation, the cells were treated with 20 µM FK. Forty-eight hours later, cell fusion was determined by immunostaining with anti-E-cadherin as indicated above, and specific gene silencing was verified by RT-PCR as described above. The primer pairs used were as follow: Human β-actin, 5′-ACAATGAGCTGCGTGTGGCT-3′ (forward) and 5′-TCTCCTTAATGTCACGCACGA-3′ (reverse); human VASH2, 5′-ACGTCTCAAAGATGCTGAGG-3′ (forward) and 5′-TTCTCACTTGGGTCGGAGAG-3′ (reverse); human Gcm-1, 5′-GCTGGGACTTGAACCAGCAGTAA-3′ (forward) and 5′-CTCAAGCACCTTGGACCAGGA-3′ (reverse); Syncytin-1, 5′-CGCCTGCTCTTCAAACAA-3′ (forward) and 5′-GGCCATGGGGATTTATGATT-3′ (reverse); Syncytin-2, 5′- TCGGATACCTTCCCTAGTGC-3′ (forward) and 5′-TGTATTCCGGAGCTGAGGTT-3′ (reverse).

For the overexpression of VASH2, BeWo cells were infected with non-proliferative adenovirus vectors encoding *human VASH2 gene* (AdVASH2) [11] or LacZ (AdLacZ) as control. Accordingly, BeWo cells were plated in 6 cm dishes at 1.5×10^5^ cells/ml. On the following day, medium was replaced by fresh ones containing AdVASH2 or AdLacZ at a final multiplicity of infection (MOI) of 10, and the cells were incubated for another 48 hours. Thereafter, cell fusion and the expression of Gcm-1, syncytin-1 and syncytin-2 were evaluated as described above.

### Calculations and statistical analysis

The data were analyzed as the mean and standard deviation, except in the case of the maternal body weight and fetus number of fetuses per dam (mean and standard error). For evaluation of the difference in placental mRNA expression between wild type and *Vash2^(−/−)^*, Welch's t test was used. The statistical significance of differences among 3 groups (WT, *Vash1^(−/−)^* and *Vash2^(−/−)^*) was evaluated by the use of Steel-Dwass test. The significance levels were taken as p<0.001, p<0.05, and p<0.01.

## Results

### Differential localization of VASH1 and VASH2 in the placenta

**Figure 1:**
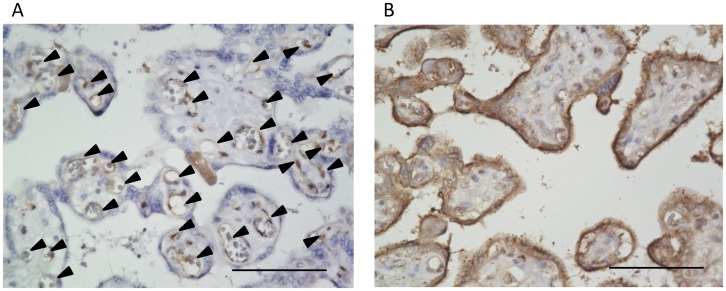
Localization of VASH1 and VASH2 in human placenta. Immunohistochemical analysis for the localization VASH1 (A) and VASH2 (B) in the human placenta was performed. Arrowheads indicate VASH1 vessels (A). Bar = 100 µm.

In order to identify the localization of VASH1 and VASH2 proteins in the placenta, we performed an immunohistochemical analysis of human placental tissue taken at term pregnancy. We previously showed the selective localization of VASH1 protein in ECs in the human placenta [7]. The present analysis confirmed this previous observation, and further revealed that its expression tended to be more intense at the villous stem (Fig. 1A). In contrast, the localization of VASH2 protein in the placenta had not been determined previously. Here we revealed for the first time that VASH2 protein was selectively localized in the trophoblasts (Fig. 1B).

### Opposing role of VASH1 and VASH2 in fetoplacental vascularization

**Figure 2:**
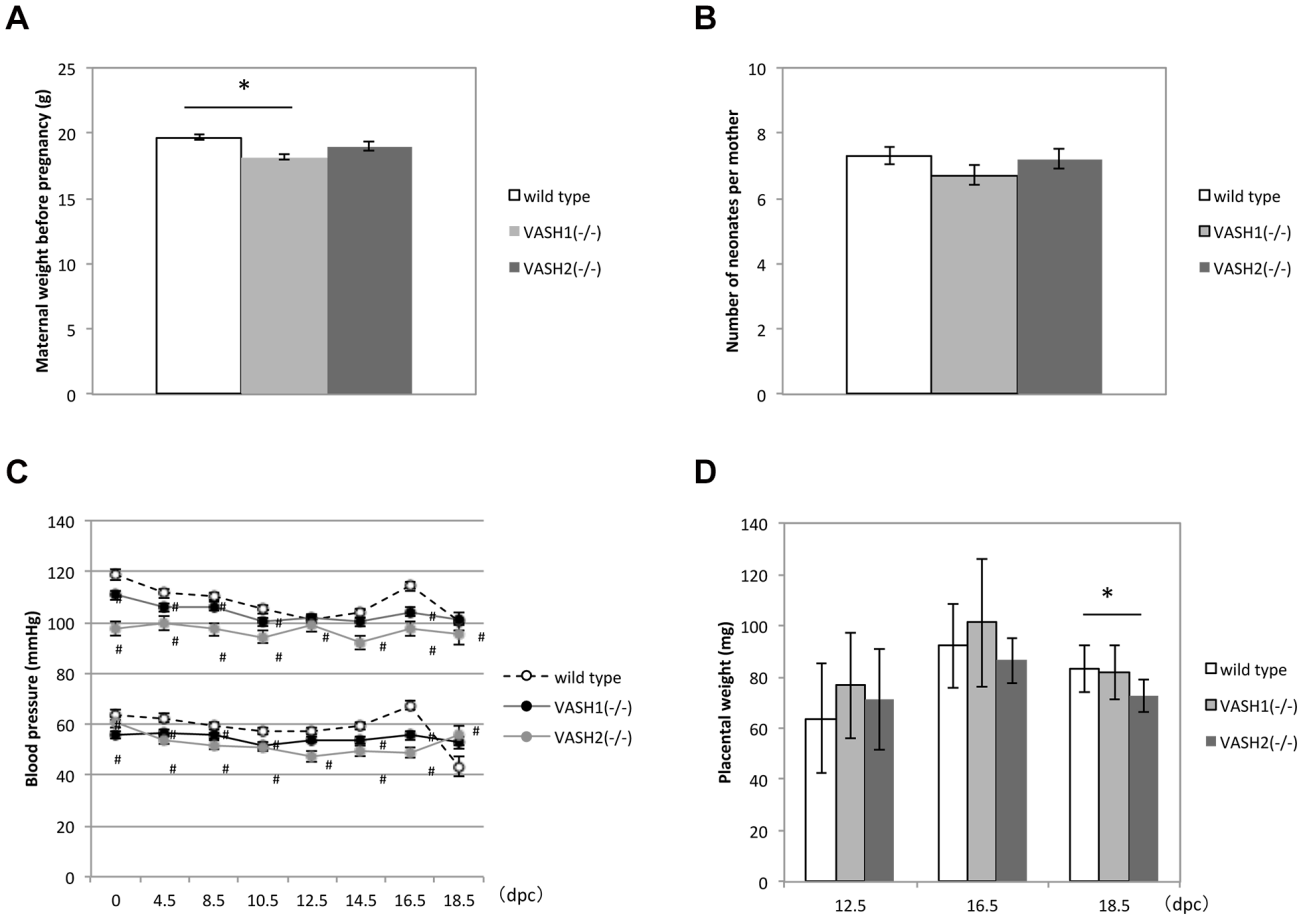
Course of pregnancy in WT, *Vash1^(−/−)^* and *Vash2^(−/−)^* mice. A: Comparison of maternal weights of WT (N = 30), *Vash1^(−/−)^* (N = 45), and *Vash2^(−/−)^* (N = 20) mice. *P<0.01. B: Comparison of number of neonates per WT (N = 32), *Vash1^(−/−)^* (N = 45), and *Vash2^(−/−)^* (N = 20) dams. C: Blood pressure of WT (N = 16), *Vash1^(−/−)^* (N = 15), and *Vash2^(−/−)^* (N = 7) dams measured at 0, 4.5, 8.5,10.5, 12.5, 14.5, 16.5, and 18.5 dpc. #P<0.05. D: Wet weight of WT (N = 37), *Vash1^(−/−)^* (N = 37), and *Vash2^(−/−)^* (N = 19) placentas. *P<0.01.

To disclose the function of VASH1 and VASH2 in the placenta, we evaluated the course of pregnancy in WT, *Vash1^(−/−)^* and *Vash2^(−/−)^* mice. The maternal weight before pregnancy (Fig. 2A) and the number of neonates per dam (Fig. 2B) were not significantly different among WT, *Vash1^(−/−)^*, and *Vash2^(−/−)^* mice. The blood pressure was low in both *Vash1^(−/−)^* and *Vash2^(−/−)^* mice (Fig. 2C). Interestingly, the weight of the placenta in *Vash2^(−/−)^* mice at 18.5 dpc was significantly lower than that in WT mice (Fig. 2D).

**Figure 3:**
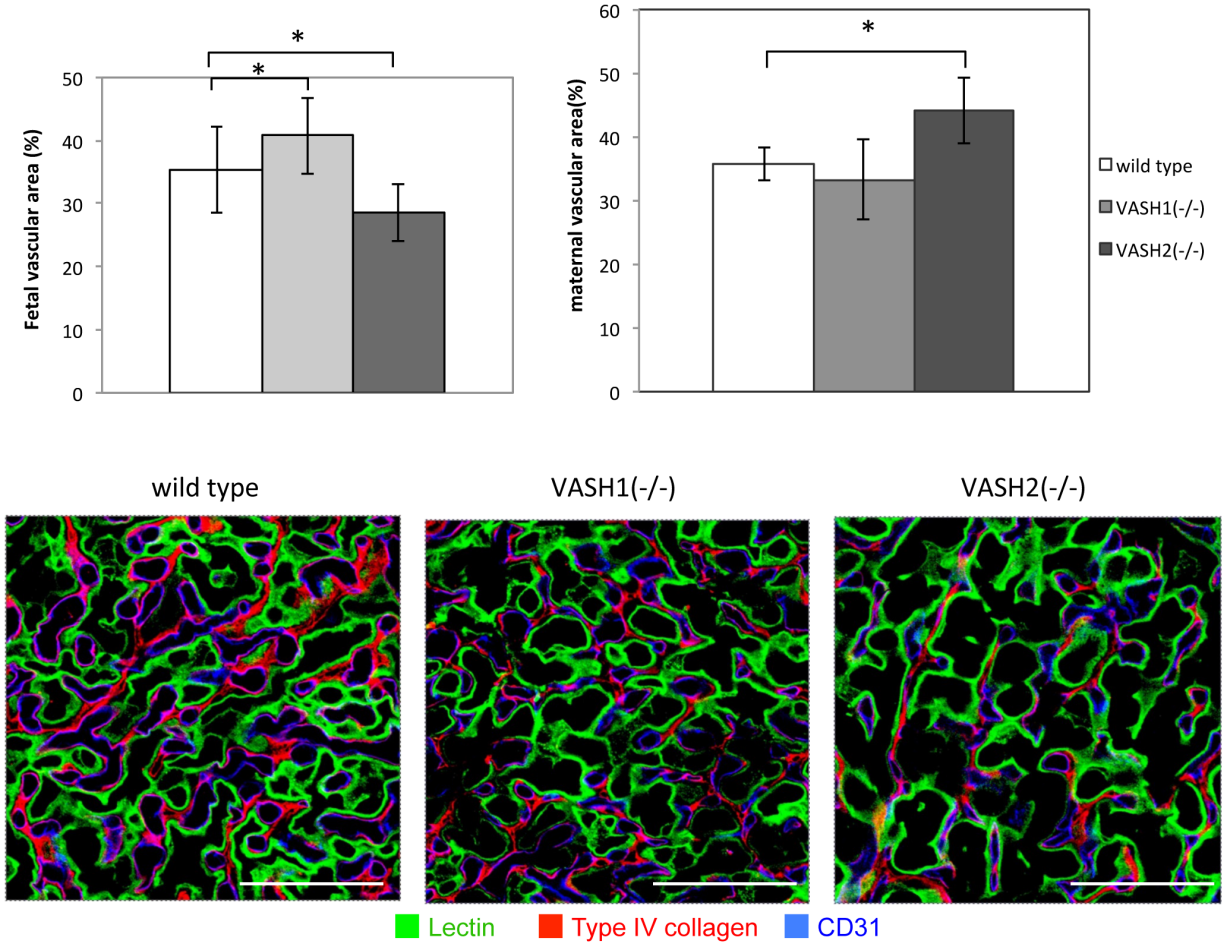
Vascularization of placenta in WT, *Vash1^(−/−)^*, and *Vash2^(−/−)^* mice. Upper panels show vascular morphogenesis. The triple staining with tomato lectin (green), anti-CD31 (blue), and anti-type IV collagen (red) was performed as described in Materials and Methods. Tomato lectin identified the maternal blood vessels; and CD31-positive structures, the fetal blood vessels. The presence of type IV collagen indicated the basement membrane. Bar = 50 µm. Lower graph on the left show the fetal vascular area, and that on the right shows the maternal vascular area determined for WT (N = 5), *Vash1^(−/−)^* (N = 3), and *Vash2^(−/−)^* (N = 3) placentas. Ten 400× fields per placenta were used for quantification. *P<0.01.

As VASH1 and VASH2 regulate angiogenesis in a contradictory manner [10], we assumed that the placental vasculature of *Vash1^(−/−)^* mice and *Vash2^(−/−)^* mice might be altered. To characterize the difference in vascular structure, we performed triple staining with tomato lectin (green) and antibodies against CD31 (blue) and type IV collagen (red). The area covered by CD31 indicated the fetal vascular area; and that by tomato lectin the maternal blood space (Fig. 3 upper panels). The fetal vascular area was significantly increased in *Vash1^(−/−)^* mice and decreased in *Vash2^(−/−)^* mice, whereas the maternal vascular area was significantly increased in *VASH2^(−/−)^* mice (Fig. 3 lower panels). We assumed that the increased maternal vascular area in *VASH2^(−/−)^* mice might be due to the poorly developed villi.

**Figure 4:**
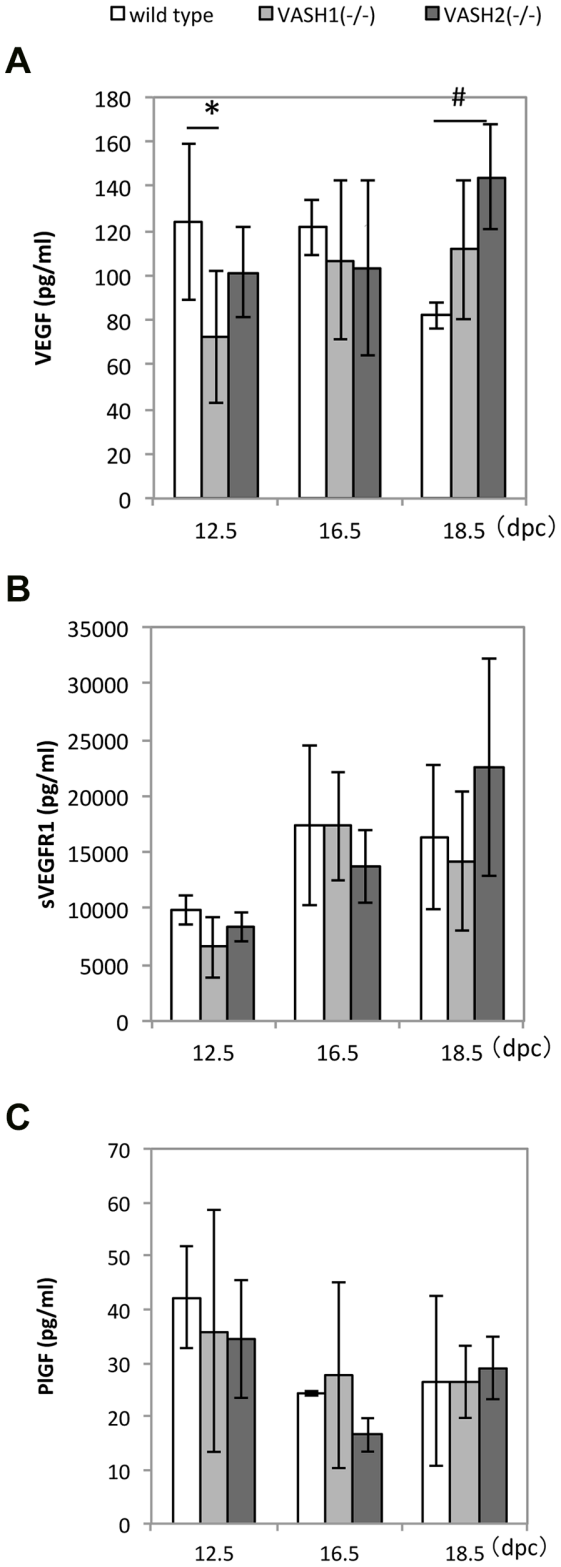
Serum levels of VEGF, sVEGFR1, and PlGF in WT, *Vash1^(−/−)^*, and *Vash2^(−/−)^* dams. A: Serum levels of VEGF-A at 12.5, 16.5, and 18.5 dpc. The respective numbers of WT dams at these time points were 6, 3 and 4; of *Vash1^(−/−)^* ones, 7, 10 and 22; and of *Vash2^(−/−)^* dams, 4, 4 and 8. ^#^P<0.05, *P<0.01. B: Serum levels of sVEGFR1 at 12.5, 16.5, and 18.5 dpc. The respective numbers of WT dams at these time points were 5, 4, and 5; of *Vash1^(−/−)^* ones, 6, 8 and 17; and of *Vash2^(−/−)^* dams, 4, 4, and 5. C: Serum levels of PlGF at 12.5, 16.5, and 18.5 dpc. Respective numbers of WT dams at these stages were 4, 2 and 2; of *Vash1^(−/−)^* ones, 5, 5, and 5; and of *Vash2^(−/−)^* dams, 3, 3, and 4.

We further determined the serum levels of VEGF-A and its related proteins, PlGF and sVEGFR1, all of which are closely associated with pregnancy. Interestingly, the serum VEGF-A was significantly low in *Vash1^(−/−)^* mice at 12.5 dpc, and was significantly high in *Vash2^(−/−)^* mice at 18.5 dpc (Fig. 4A). We could not find any significant differences in the serum levels of sVEGFR1 and PlGF (Fig. 4B and C).

### VASH2 expressed in trophoblasts regulates cell fusion for syncytiotrophoblast formation

**Figure 5:**
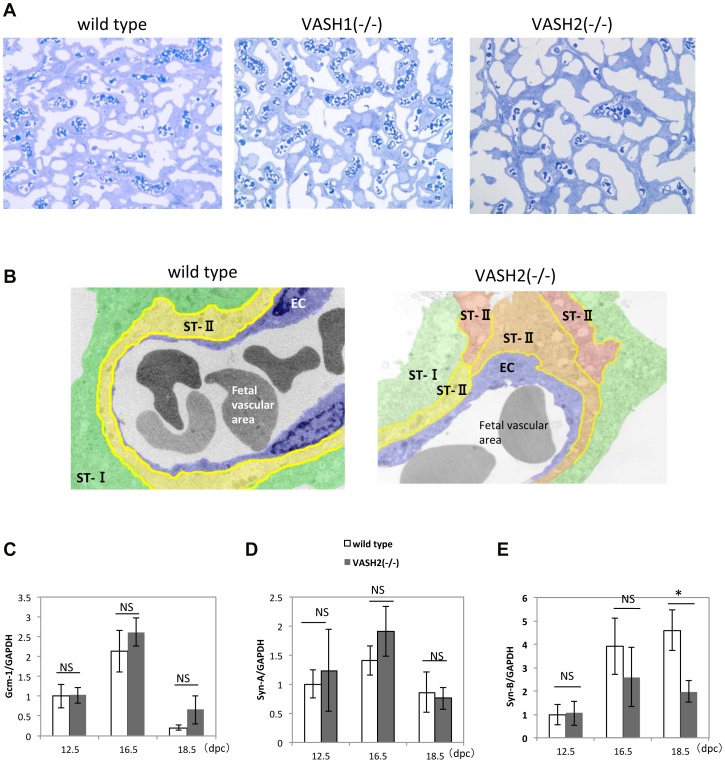
Labyrinth and syncytiotrophoblast layers of WT, *Vash1^(−/−)^* and *Vash2^(−/−)^* mice. A: Semi-thin sections of the labyrinth layer of WT, *Vash1^(−/−)^*, and *Vash2^(−/−)^* placentas. Bar = 20 µm. B: Electron microscopic pictures of WT and *Vash2^(−/−)^* placentas. Purple indicates ECs; green, ST-I; and yellow, ST-II. Bar = 5 µm. C–E: Expression of Gcm-1, Syn-B, and Syn-A in WT, *Vash1^(−/−)^* and *Vash2^(−/−)^* placentas at the indicated dpc, was determined by qRT-PCR. At 12.5, 16.5, and 18.5 dpc, the respective placenta numbers were 7, 6, and 5 for WT; 7, 7, and 7 for *Vash1^(−/−)^*; and 5, 7, and 6 for *Vash2^(−/−)^*. *P<0.01, NS; not significant.

We further clarified the morphological changes in the placenta of the 3 types of mice. Electron microscopic observation of semi-thin sections showed that the fetal vascular area containing red blood cells within the labyrinth layer was increased in *Vash1^(−/−)^* mice and decreased in *Vash2^(−/−)^* mice (Fig. 5, A). These observations correlated well with the immunohistochemical findings shown in Fig. 3. However, a more striking change that we noticed was the thin and poorly developed villi in the *Vash2^(−/−)^* mice. As a result, the placenta was more porous having larger maternal lacunae in the *Vash2^(−/−)^* mice. This may explain the reduced weight of placenta in the *Vash2^(−/−)^* mice (Fig. 2D). The mouse placenta has 2 distinct syncytiotrophoblast layers, ST-I and ST-II. Our careful observation by the electron microscopy revealed that the cell fusion of ST-II was incomplete in the *Vash2^(−/−)^* mice (Fig. 5B). Murine endogenous retrovirus (ERV) envelope glycoprotein syncytin-A (Syn-A) and syncytin-B (Syn-B) are expressed in trophoblasts and regulates their fusion [15], [16]. The transcription factor glial cells missing-1 (Gcm-1) regulates the expression of Syn-A [17]. We therefore compared the mRNA expression of Gcm-1, Syn-A, and Syn-B in the placenta, and found that only the expression of Syn-B was down-regulated in the *Vash2^(−/−)^* mice (Fig. 5 C–E).

**Figure 6:**
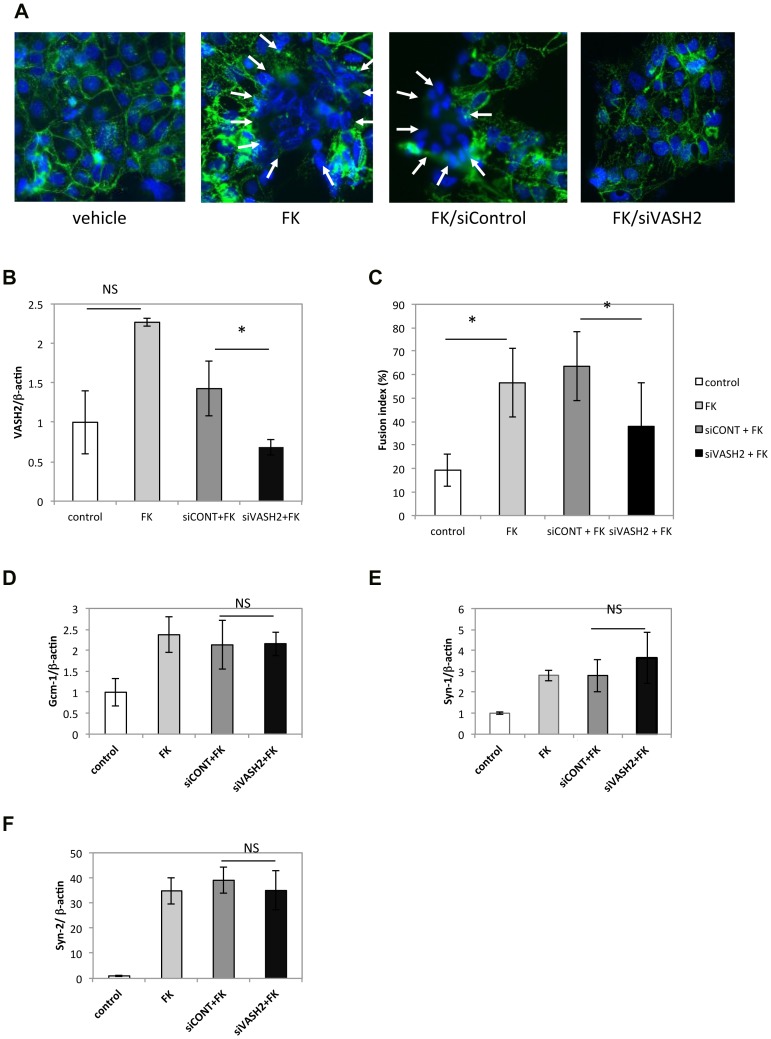
Knockdown of VASH2 inhibited the forskolin-induced fusion of BeWo cells. A: BeWo cells with or without siRNA treatment were stimulated with FK, and cell fusion was observed as described in Materials and Methods. Bar = 100 µm. Arrows indicate fused cells with multiple nuclei. B: Expression of human VASH2 was quantified (N = 3). *P<0.01, NS; not significant. C: Cell fusion was quantified as described in Materials and Methods (N = 2, 10 fields each). *P<0.01. D–F: Expression of Gcm-1, Syn-2, and Syn-1 in BeWo cells with each treatment (N = 3) was determined by qRT-PCR. NS; not significant.

**Figure 7:**
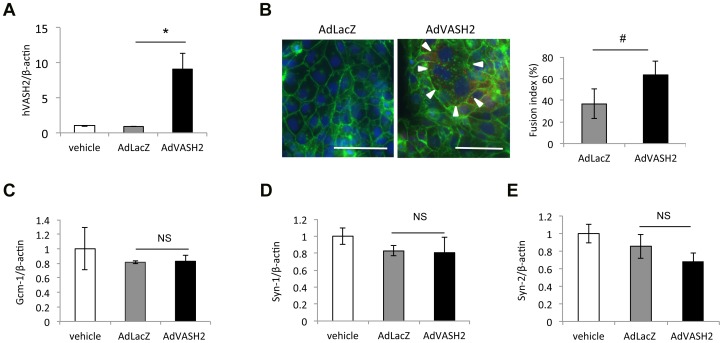
The overexpression of VASH2 augmented the fusion of BeWo cells. A: BeWo cells were infected with adenovirus vectors. Expression of human VASH2 was quantified by qRT-PCR (N = 3). B: Cell fusion was observed as described in Materials and Methods. Bar = 100 µm. Arrowheads indicate a fused cell with multiple nuclei. Cell fusion was quantified as described in Materials and Methods (N = 3, 5 fields each). #<P0.05. C: Expression of Gcm-1 was quantified by qRT-PCR (N = 3). D: Expression of Syn-1 was quantified by qRT-PCR (N = 3). E: Expression of Syn-2 was quantified by qRT-PCR (N = 3). NS; not significant.

To examine the role of VASH2 more directly, we used BeWo cells a human trophoblastoid choriocarcinoma cell line, in culture. As it is previously reported [18], [19], the FK treatment induced cell fusion in BeWo cells (Fig. 6A) When the VASH2 expression in BeWo cells was knocked-down by siRNA (Fig. 6B), we observed a significant decrease in cell fusion (Fig. 6A and C). Human ERV envelope glycoproteins, Syn-1 and Syn-2 have been identified to regulate the fusion of trophoblasts [18]–[22]. They are not orthologous to murine Syn-A and Syn-B, but Gcm-1 regulates the expression of Syn-1 [19]. We therefore examined the expression of Gcm-1, Syn-1 and Syn-2 in BeWo cells, and found that it was not altered by the knock-down of VASH2 (Fig. 6D–F). To further clarify the role of VASH2 in the cell fusion, we overexpressed human VASH2 in BeWo cells. AdVASH2 infection significantly increased the expression of VASH2 and cell fusion of BeWo cells (Fig. 7A and B). Importantly, the AdVASH2 infection did not alter the expression of Gcm-1, Syn-1 and Syn-2 in BeWo cells (Fig. 7 C–E).

## Discussion

The vasohibin family comprises 2 proteins, the anti-angiogenic VASH1 and the pro-angiogenic VASH2. It was previously shown that the VASH1 protein is selectively localized in ECs at the site of angiogenesis associated with various pathophysiological conditions and at that occurring in the human placenta [7], [23]–[28]. Here we confirmed its selective localization in ECs in the placenta. The localization of VASH2 protein in the placenta had not been previously examined. Our present study revealed for the first time that VASH2 was selectively localized in trophoblasts in the placenta. The distinctive expression and localization of VASH2 from those of endothelial VASH1 were shown previously. For example, VASH2 is expressed in infiltrating CD11b^+^ monocyte/macrophage lineage cells that infiltrate the dermis in hypoxia-induced angiogenesis [11]. In addition, VASH2 is expressed in ovarian or hepatocellular carcinoma cells [29], [30]. The present study disclosed another instance of such distinctive localization of VASH2 from that of endothelial VASH1, i.e., in the placenta.

When we evaluated the role of VASH1 and VASH2 in the vascularization of placenta, our analysis revealed that the fetal vascular area was significantly increased in the *Vash1^(−/−)^* mice and decreased in the *Vash2^(−/−)^* ones. These results correlate well with the anti-angiogenic function of VASH1 and pro-angiogenic function of VASH2. Moreover, their differential expressions further suggest that VASH1 acts as an autocrine factor whereas VASH2 acts as a paracrine factor. Interestingly, the serum level of VEGF-A was significantly low in *Vash1^(−/−)^* mice at E12.5, and significantly high in *Vash2^(−/−)^* mice at E18.5. We assumed that those changes in VEGF-A were due to biological adaptation; i.e., when the angiogenesis inhibitor VASH1 was defective, angiogenic VEGF-A was decreased, and when angiogenesis stimulator VASH2 was defective, angiogenic VEGF-A was increased. These adaptations would be expected to minimize the change of vascularization in *Vash1^(−/−)^* and *Vash2^(−/−)^* mice.

Here we disclosed the novel function of VASH2, namely, its involvement in trophoblast fusion. A neutralizing anti-human VASH2 monoclonal antibody inhibited the FK-induced fusion of HUVECs (Suenaga et al. unpublished observation). Thus, this effect of VASH2 is mediated via the autocrine/paracrine manner. The syncytiotrophoblast layer is formed by the fusion of trophoblasts. The mouse placenta has 2 distinct and highly specialized syncytiotrophoblast layers, ST-I and ST-II; whereas the human placenta has a single layer. The known regulators of trophoblast fusion are ERV envelope glycoproteins, Syn-A and Syn-B in mice and Syn-1 and Syn-2 in humans [15]–[22]. Among them, Syn-A and Syn-1 are the target of Gcm-1. We observed that *Vash2^(−/−)^* mice displayed impaired cell fusion of trophoblasts only in ST-II. The defect in trophoblasts in *Vash2^(−/−)^* mice might not severely affect the function of placenta, because the number of neonates was not impaired in *Vash2^(−/−)^* mice (Fig. 2B).

This defect of ST-II resembles that of *Syn-B^(−/−)^* mice [31]–[33]. Interestingly, the expression of Syn-A was unchanged but that of Syn-B was down-regulated in the *Vash2^(−/−)^* placenta. These observations may explain the reason why the impaired cell fusion was only observed in ST-II of the *Vash2^(−/−)^* placenta, and thus suggest a possible interaction between VASH2 and Syn-B for the formation of ST-II in mice. Nonetheless, the interaction of VASH2 and ERV envelope glycoproteins cannot be applied to the human system. FK induced the fusion of human BeWo cells, and this fusion was inhibited by the knockdown of VASH2. In this situation, the expression of Gcm-1, Syn-1, and Syn-2 was unchanged. Conversely, the overexpression of VASH2 by the AdVASH2 infection augmented the fusion of BeWo cells without any changes in the expression of Gcm-1, Syn-1, and Syn-2. Thus, the role of VASH2 in the fusion of trophoblasts is independent of Gcm-1 and ERV envelope glycoproteins at least in the human system.

Cell fusion is a phenomenon that is seen not only in placentation but also in various physiological/pathophysiological conditions such as fertilization, development of skeletal muscle and bone, removal of apoptotic cells by macrophage, and the development and progression of cancers; but the commonality of the mechanism of cell fusion in those conditions is currently unknown [34], [35]. The expression of VASH2 is scarce and limited, but our earlier observations showed this expression in cancer cells [28], [29] and cells of the macrophage lineage [11]. The present study revealed its expression in trophoblasts. We also have detected the expression of VASH2 in skeletal muscle (unpublished data). These circumferential lines of evidence may suggest a role for VASH2 in cell fusion. This hypothesis needs to be investigated in a future study.

In summary, we disclosed the role of vasohibin family proteins in placental morphogenesis. As expected, VASH1 in ECs acted as an angiogenesis inhibitor and VASH2 in trophoblasts acted as an angiogenesis stimulator, in the placenta. However, perhaps the most intriguing finding was that VASH2 in trophoblasts played the role in the cell fusion for syncytiotrophoblast formation. Our present study provides innovative information on the function of VASH2 beside the stimulation of angiogenesis. Further study is currently underway to clarify the mechanism as to how VASH2 regulates the fusion of trophoblasts
